# Aerobic dissimilatory nitrate reduction to ammonium in *Streptomyces hydrogenans* EM-F5: metabolic insights, physiological activity, and driving factors

**DOI:** 10.1128/aem.01039-26

**Published:** 2026-08-26

**Authors:** Manman Zhang, Jiachen Wang, Dandan Li, Tengxia He, Pan Wu

**Affiliations:** 1 Key Laboratory of Plant Resource Conservation and Germplasm Innovation in Mountainous Region (Ministry of Education), Guizhou Key Laboratory of Agricultural Microbiology, College of Life Sciences, Guizhou University 377868, Guiyang, Guizhou Province, China; 2 Key Laboratory of Karst Georesources and Environment (Guizhou University), Ministry of Education, Guizhou University 655803, Guiyang, Guizhou Province, China; Colorado School of Mines, Golden, Colorado, USA

**Keywords:** aerobic DNRA, actinomycetes, ammonium production

## Abstract

Dissimilatory nitrate reduction to ammonium (DNRA) can retain nitrogen in the soil and reduce greenhouse gas emissions. However, bacterial anaerobic DNRA has certain drawbacks, such as the requirement for strictly anaerobic conditions, complex management, and high cost. A new actinomycete strain EM-F5 was isolated and identified as *Streptomyces hydrogenans*. Strain EM-F5 showed broad environmental adaptability (pH 6.20–9.20, 0.10–0.50 × 10^8^ CFU/mL of inoculum size, 3.30–7.25 mg/L of dissolved oxygen, and 18℃–38℃). Using starch as the carbon source, the strain EM-F5 achieved 98.04% nitrate reduction and 54.89% ammonium production efficiency via aerobic DNRA, whereas only 37.35% of nitrate was converted under anaerobic conditions. Electron transport system activity (ETSA), adenosine triphosphate (ATP), and nicotinamide adenine dinucleotide (NADH/NAD^+^) reached up to 0.11 μgO_2_g^−1^min^−1^, 29.73 μmol/g, and 5.24, respectively, confirming the high efficiency of aerobic DNRA. The specific activity of nitrate reductase (NR) and nitrite reductase (NIR) was detected as 0.323 and 0.018 U/mg protein, respectively, confirming its key catalytic function in the DNRA pathway. Nitrogen balance (nitrogen loss of 25.46%) experiments further confirmed the aerobic DNRA pathway of strain EM-F5 was NO_3_^−^-N→NO_2_^−^-N→NH_4_^+^-N. These findings expand the current understanding of DNRA in actinomycetes and provide a promising microbial resource for nitrogen recycling in soil.

**IMPORTANCE:**

Strain EM-F5 effectively performed dissimilatory nitrate reduction to ammonium (DNRA) with starch as the carbon source under aerobic conditions. Nitrate reduction was as high as 98.04%, and the ammonium production efficiency accounted for 54.89% of the total converted nitrate nitrogen. The aerobic DNRA pathway of strain EM-F5 was NO_3_^−^-N→NO_2_^−^-N→NH_4_^+^-N. Meanwhile, it converted 37.35% of nitrate in anaerobic environments. So far, there have been few studies that have demonstrated that actinomycetes can efficiently perform DNRA under aerobic conditions and also convert nitrate under anaerobic conditions. This provides a superior microbial source for treating highly nitrate-contaminated soil.

## INTRODUCTION

Nitrogen is a vital nutrient for all living organisms, with soil serving as the primary source of nitrogen for crops. However, natural nitrogen fixation is insufficient to meet the escalating demands of food production driven by rapid global population growth. As a result, approximately half of the world’s food supply now relies on the application of industrial fertilizers to agricultural lands. The excessive application of fertilizers leads to the accumulation of surplus nitrogen in the soil, which is subsequently converted into nitrate. This nitrate can then be mitigated through biological processes such as denitrification and dissimilatory nitrate reduction to ammonium (DNRA). DNRA reduces nitrate to ammonium, whereas denitrification permanently removes nitrate from the ecosystem in the form of nitrogen gas (1). Denitrification has attracted considerable attention in recent decades because it can cause nitrogen loss and increase the risk of greenhouse gas emission. Sustainable nitrogen utilization and environmental protection require effective nitrate management. Therefore, controlling nitrate conversion and enhancing nitrogen retention in the environment are of great importance.

DNRA is a method for converting nitrate/nitrite to ammonium, which is a crucial component of the nitrogen recovery strategy (2). The conventional view is that DNRA processes are performed only by bacteria in anaerobic or oxygen-limited environments (such as sediments, silt, or anoxic aquatic biological systems) (3). For example, *Desulfovibrio* sp. CMX was capable of converting 0.345 g/L of nitrate into 0.207 g/L of ammonium during a 72-h anaerobic culture period, corresponding to ammonium production efficiency of 60% (4). In anaerobic environments, the *Desulfovibrio vulgaris* strains AL-1, AL-2, 37-2 and 43-2 converted 32.7%–46% of nitrate (100 mg/L) into ammonium during incubation for 21 days (5). Although DNRA is possible under aerobic conditions, most nitrate or nitrite conversion is incomplete or the ammonium production efficiency is very low. For instance, *Pseudomonas putida* Y-9 could transform 88.47% of nitrate (116.63→13.45 mg/L) after 36 h, but only 3.42 mg/L ammonium was produced through DNRA under aerobic conditions (6). Further investigation of the capabilities of other microorganisms to perform DNRA under aerobic conditions is essential, which can provide a high-quality bacterial source for nitrogen retention in soil. Numerous actinomycetes have been isolated from biogas digesters and sludge. Examples included *Gordonia* sp. TD-4, *Thermomonospora*, *Micromonospora*, *Streptomyces*, and *Streptomyces mediolani* EM-B2. Actinomycetes exhibit certain advantages for DNRA, for example, (i) actinomycetes are the principal genera for DNRA in compost, wetlands, and Antarctica, thus showing great application potential (7, 8); (ii) actinomycetes can perform DNRA at a high composting temperature (9); and (iii) actinomycetes have the potential to produce ammonium efficiently without the accumulation of harmful intermediates. For instance, *Streptomyces mediolani* EM-B2 produces as much as 31.61 mg/L of ammonium during simultaneous nitrification and denitrification without nitrite accumulation (10). Therefore, actinomycetes can overcome the limitations of low ammonium production rate, anaerobic reaction conditions, and complex management of most DNRA microorganisms.

The DNRA process may be affected by many factors, such as electron donor type, iron, pH, and sulfides, which can determine the relative proportions of DNRA and denitrification (11). The DNRA process may benefit from the abundant availability of organic electron donors (12). A high organic carbon/nitrate ratio is generally favorable for nitrate distribution to DNRA (13). Fe^2+^ promotes DNRA activity in bacterial communities in a reactor (14). Several studies have shown that pure cultures of actinomycetes can perform denitrification and DNRA under anaerobic and aerobic conditions. Thus, the factors influencing DNRA by actinomycetes require further exploration.

*Streptomyces hydrogenans*, an actinomycete, has been reported to promote organic matter degradation in wastewater through its metabolic activity when immobilized on biochar (15). *Streptomyces hydrogenans* can be applied as a growth promoter, photosynthetic pigment enhancer, and biological control agent for *Solanum lycopersicum* plants (16). In addition, *Streptomyces hydrogenans* can produce a pectinase to generate unsaturated oligogalacturonic acids, which can be used as an antioxidant and anticarcinogen (17). To date, the ability of *Streptomyces hydrogenans* to produce ammonium by dissimilatory nitrate/nitrite reduction has not been reported. In this study, a new actinomycete strain, EM-F5, was isolated and screened from the Caohai Wetland, China, using nitrate as the nitrogen source. The strain EM-F5 was identified by morphological observation and 16S rRNA gene sequencing. The characteristics and differences in DNRA under anaerobic and aerobic conditions were investigated at the metabolic level. The influence of environmental factors (temperature, pH, inoculum amount, dissolved oxygen, and Fe^2+^/NO_3_^−^, S^2−^/NO_3_^−^, and NO_2_^−^/NO_3_^−^ ratios) on DNRA by strain EM-F5 was also evaluated. Finally, the determination of key enzyme activity and nitrogen balance further confirmed the high efficiency of the aerobic DNRA process. These findings uncover the dual role of actinomycetes in aerobic DNRA and anaerobic nitrate conversion, distinguishing their differential performance under varying oxygen conditions.

## MATERIALS AND METHODS

### Strain source

Actinomycetes strains were isolated from Caohai Wetland in Weining County, Guizhou Province.

### Medium

Enrichment medium (Gao’s No. 1 medium, 1 L, pH = 7.40) consists of the following: 20.0 g soluble starch, 1.0 g KNO_3_, 0.01 g FeSO_4_·7H_2_O, 0.50 g K_2_HPO_4_, 0.24 g MgSO_4_, 0.50 g NaCl, and 0.05 g K_2_Cr_2_O_7_. This medium was selected to isolate actinomycetes.

Luria–Bertani (LB) liquid medium (1 L, pH = 7.20) consists of the following: 10.0 g sodium chloride, 10.0 g tryptone, and 5.0 g yeast extract. This medium was used for activation and expanded culture of isolated strains. The solid medium was prepared by adding 18% agar to the liquid medium and used for the isolation and purification of strains.

DNRA medium (1 L, pH = 7.20) consists of the following: 0.72 g KNO_3_, 3.0 g soluble starch, 0.01 g Fe_2_(SO_4_)_3_, 0.04 g MgSO_4_, 3.50 g K_2_HPO_4_, 1.50 g KH_2_PO_4_, 0.01 g CaCl_2_, 0.002 g CuSO_4_, 0.002 g ZnSO_4_, and 0.001 g MnSO_4_.

The media were sterilized at 121℃ and 0.11 MPa for 30 min.

### Isolation, screening, and molecular identification of actinomycetes

One gram of the soil sample was cultured for 5 days at 28 °C with shaking at 150 rpm in 100 mL enrichment medium, then 1 mL of the culture medium was transferred to fresh medium, and the same culture protocol was repeated two or three times. The bacterial mycelium was spread on LB solid medium and incubated at 28℃. Single colonies were selected and purified by repeated streaking on fresh agar plates two or three times. The primary selection criteria were based on two key metabolic capabilities: the efficiency of nitrate conversion from the culture medium and the subsequent production of ammonium as an end product. Consequently, the strains exhibiting the most superior performance in these two metrics were designated as the lead candidate strains for further investigation into the DNRA pathway. The strains were stored in 30% glycerol solution at −20℃ and −80℃. The strain morphology was observed after Gram staining with an Olympus BX53-DIC bright-field microscope (Tokyo, Japan) and with an SU8100 scanning electron microscope (Hitachi, Japan).

Genomic DNA of the candidate strains was extracted using the Chelex-100 method. One milliliter of the bacterial suspension in LB liquid medium was centrifuged (7,000 rpm, 5 min) in a 2-mL EP tube, then washed with 500 µL sterilized ultrapure water, and centrifuged at 13,500 × *g* for 1 min. The supernatant was discarded, and the bacteria were collected. Add 4–5 capsules of 5% Chelex-100 reagent and thoroughly mix with bacteria. Then it was boiled at 100℃ for 10 min, cooled to room temperature, and centrifuged at 13,500 × *g* for 5 min. The supernatant was used for PCR amplification (CFXConnect&T100, America) of the 16S rRNA gene with the universal primers 27F and 1492R (18). The PCR (25 µL) protocol followed the method of Zhang et al. (19). After gel electrophoresis, the PCR products were submitted to the Shanghai Shengong Biotechnology Co., Ltd., for molecular cloning and sequencing. MEGA 7 was used to generate a multiple sequence alignment. A dendrogram was constructed using the neighbor-joining method for identification of the candidate strains.

### Assessment of actinomycete DNRA capability under aerobic and anaerobic conditions

LB liquid medium was supplemented with 1 mL glycerol-preserved bacterial suspension, which was activated for 36 h at 28°C with shaking at 150 rpm. The bacteria were collected and washed in sterilized ultrapure water two or three times. An approximate concentration of 0.006 g/L (initial OD_600_ = 0.3 × 10^8^ CFU/mL) wet mycelium was inoculated into DNRA medium and cultured at 28°C and 150 rpm. The concentrations of nitrate, nitrite, and hydroxylamine; the pH (DDS-307A conductivity meter); and fresh weight in the medium were detected at 48-h intervals to assess the DNRA activity. Anaerobic cultures were performed in 250-mL bottles containing 100 mL of DNRA medium, following the same protocol as aerobic cultures except for the initial nitrogen purging (15 min). After inoculation, the bottles were incubated at 28°C and 150 rpm, and DNRA activity was monitored at 48-h intervals. All experiments were repeated three times with uninoculated strains used as the control group. The formula C1 (%) = (R1 − R2)/R1 ×100% was used to calculate the nitrate conversion efficiency, and A1 (%) =A/(R1 − R2) ×100% was used to calculate the ammonium production efficiency, where C1, A1, A, R1, and R2 were the nitrate conversion efficiency, ammonium production efficiency, ammonium production concentration, the initial nitrate concentration, and the terminal nitrate concentration, respectively.

### Assessment of the effect of environmental factors on aerobic DNRA

The effects of temperature, dissolved oxygen, pH, and inoculum concentration on DNRA activity were assessed using a single-factor test. The strain EN-F5 was enriched and activated in LB liquid medium at 25℃ and 150 rpm for 36 h. To collect the cells, the culture suspension was centrifuged at 7,000 rpm for 5 min and then washed twice with sterile pure water. The resuspended cells were quantitatively inoculated into a 250-mL sterilized conical flask containing 100 mL sterile synthetic wastewater. The optimized basic medium contained KNO_3_ (0.72 g/L), soluble starch (3.0 g/L), Fe_2_(SO_4_)_3_ (0.01 g/L), MgSO_4_ (0.04 g/L), K_2_HPO_4_ (3.50 g/L), KH_2_PO_4_ (1.50 g/L), CaCl_2_ (0.01 g/L), CuSO_4_ (0.002 g/L), ZnSO_4_ (0.001 g/L), and MnSO_4_ (0.001 g/L). Based on previous reports, the optimization conditions were set as follows: temperature (18, 23, 28, 33, and 38℃), dissolved oxygen (2.39, 3.30, 5.61, 7.25, and 6.85 mg/L), pH (5.20, 6.20, 7.20, 8.20, and 9.20), and inoculum size (0.10, 0.20, 0.30, 0.40, and 0.50 × 10^8^ CFU/mL). The concentrations of nitrate, nitrite, hydroxylamine, and ammonium in the medium and the growth of the strain were assessed at the initiation and conclusion of the culture period to evaluate the DNRA capability of the strain EM-F5.

Sulfide, iron, and nitrite can influence both DNRA activity and ammonium accumulation. Therefore, to evaluate whether these factors affected the aerobic DNRA activity of the isolated actinomycete strains, the Fe^2+^/NO_3_**^−^** ratio was set as 1/1, 2/1, 4/1, 6/1, or 8/1; the S^2^**^−^** /NO_3_**^−^** ratio was 0.5/1, 1/1, 2/1, 4/1, or 8/1; and the NO_2_^−^/NO_3_^−^ ratio was 1/1, 1/2, 1/4, 1/8, or 1/16. The total nitrogen concentration was unchanged. To examine further the effect of the NO_3_^−^/NO_2_^−^ ratio on the strain DNRA activity, the ratio was set as 1/9, 3/7, 5/5, 7/3, or 9/1 with changed total nitrogen. Above all, all experiments were repeated three times and no bacteria were added as the control group. The experimental protocol and culture conditions were as described directly above.

### Determination of physiological indicators in aerobic DNRA by strain EM-F5

The assay for electron transport system activity (ETSA) is based on the reduction of iodonitrotetrazolium chloride (INT) to INT-formazan during electron transfer. The intensity of the resulting color change, measured spectrophotometrically, reflects the ETSA level. A 2-mL aliquot of 0.2% INT solution was mixed with 1 mL of the culture sample collected at different time points. The mixture was incubated in the dark at 25 °C for 25 min, after which the reaction was terminated by adding 200 μL of methanol. Following centrifugation at 10,000 r/min for 5 min, the pellet was resuspended in 4 mL of 96% methanol to extract the formazan. After another centrifugation under the same conditions to remove the insoluble material, the absorbance of the supernatant was measured at 495 nm. The absorbance at 495 nm was inserted into the following equation to calculate ETSA (in μg O₂ g⁻¹ min⁻¹) = (Abs × V × 32) / (15.9 × 2 × S × T). Here, V is the resuspension methanol volume (4 mL), Abs is the 495-nm absorbance, T is the reaction time (20 min), and S is the sample volume (1 mL) (20). Adenosine triphosphate (ATP) levels were quantified using a commercial ATP Content Assay Kit (COMIN, Suzhou, China). Similarly, the concentrations of nicotinamide adenine dinucleotide (NADH and NAD^+^) were determined with an NAD(H) Content Detection Kit (WST colorimetric method) (Solarbio, Suzhou, China).

### Determination of aerobic DNRA-related enzyme activities and nitrogen balance of strain EM-F5

Following the washing and resuspension procedures described in “Assessment of actinomycete DNRA capability under aerobic and anaerobic conditions,” above, the bacterial suspension was incubated at 28 °C with shaking at 150 r/min for 144 h. After cultivation, the samples were collected and centrifuged at 7,000 r/min for 5 min at 28 °C. Cells were resuspended in an extraction buffer and disrupted using an ultrasonic cell disruptor under the following conditions: 300 W power, with a cycle of 3 s pulse on and 7 s pulse off, for a total duration of 3 min. After ultrasonication, the lysate was centrifuged at 10,000  ×  *g* for 10 min at 4 °C. The resulting supernatant was collected as the crude enzyme extract. The protein concentration of the extract was determined using a BCA protein assay kit (Solarbio). The 20 mL reaction system for determining nitrate reductase (NR) activity contained a reaction mixture of nitrate nitrogen (20 mg/L), crude enzyme extract, NADH (0.2 mmol/L), and Tris-HCl (10 mmol/L). The reduction in nitrate nitrogen content was used as an indicator for detecting NR activity. Nitrite reductase (NIR) activity was measured with a commercial nitrite reductase activity assay kit (COMIN), where the nitrite content served as an indicator of enzymatic activity. A control reaction without the enzyme extract was included in parallel. Specific activity (U/mg protein) was defined as the amount of the enzyme required to convert 1 μmol of the substrate per minute and was calculated by dividing this activity value by the protein concentration in the reaction system.

Cells were precultured in LB medium at 28°C, washed, and then transferred to media containing either malate or starch as the sole carbon source. At 0 h, one 6-mL aliquot was centrifuged (7,000 rpm, 28°C, 5 min) to collect the supernatant for determination of initial ammonium, hydroxylamine, nitrate, nitrite, and total nitrogen (initial TN₁). A second 6-mL aliquot was ultrasonically disrupted (Scientz-IID, 300 W, 15 min, and 3 s on/off), and the supernatant was filtered (0.22 µm) for measurement of total nitrogen after cell lysis. After 72 h and 168 h of cultivation, the same procedures were repeated to collect supernatants for residual nitrogen species and total nitrogen (initial TN₂), as well as disrupted cell samples for final total nitrogen (final TN₂). Nitrogen balance calculations were performed following the method of Zhang et al. (21).

### Statistical analysis and data visualization

The concentrations of nitrate, nitrite, hydroxylamine, and ammonium were detected using the Chinese national standard methods (22) (Table 1). Microsoft Excel and IBM SPSS Statistics software were used for statistical analysis of the data. The data were visualized using MEGA 7.0 and Origin 2021 software. All data are expressed as mean ± standard deviation.

**TABLE 1:** Methods for the determination of inorganic nitrogen

Inorganic nitrogen	Method
Ammonium	Indophenol blue
Hydroxylamine	8-hydroxyquinoline UV spectrophotometry
Nitrate	Ultraviolet spectrophotometry
Nitrite	N-(1-naphthyl)-ethylenediamine photometric

## RESULTS AND DISCUSSION

### Strain morphology and molecular identification

**Fig 1:**
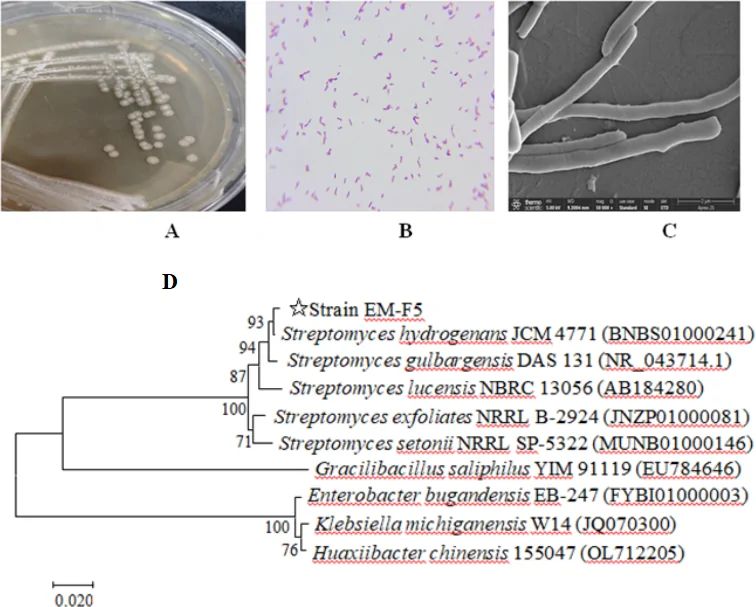
Colony morphologies and phylogenetic tree of the strain EM-F5. (**A**) Colonies on the LB plate. (**B**) Gram staining under an optical microscope. (**C**) Cell shape by the scanning electron microscope. (**D**) Phylogenetic tree of the strain EM-F5 and other related sequences based on the 16S rRNA gene sequence.

In the preliminary screening stage, actinomycetes were isolated from soil samples collected from Caohai Wetland using Gao’s No. 1 medium. The target strain EM-F5 was isolated and purified according to the selection criteria of high efficiency of nitrate conversion and high accumulation of ammonium. The colony morphology of strain EM-F5 was white, with regular edges, round, dry surface, white powder, and opaque on LB solid medium (Fig. 1A and C). The strain EM-F5 was a gram-positive bacterium (Fig. 1B). It was observed with a scanning electron microscope to form long filamentous rods, consistent with those of actinomycetes. A BLAST comparison revealed that the strain EM-F5 showed highest sequence similarity with *Streptomyces hydrogenans* JCM 4771 (99.72%). A neighbor-joining dendrogram constructed from an alignment of the nucleotide sequences of strain EM-F5 and other actinomycete strains further indicated that strain EM-F5 was most similar to *Streptomyces hydrogenans* (Fig. 1D).

### Differences in DNRA activity of strain EM-F5 under aerobic and anaerobic conditions

#### DNRA activity under aerobic conditions

**Fig 2:**
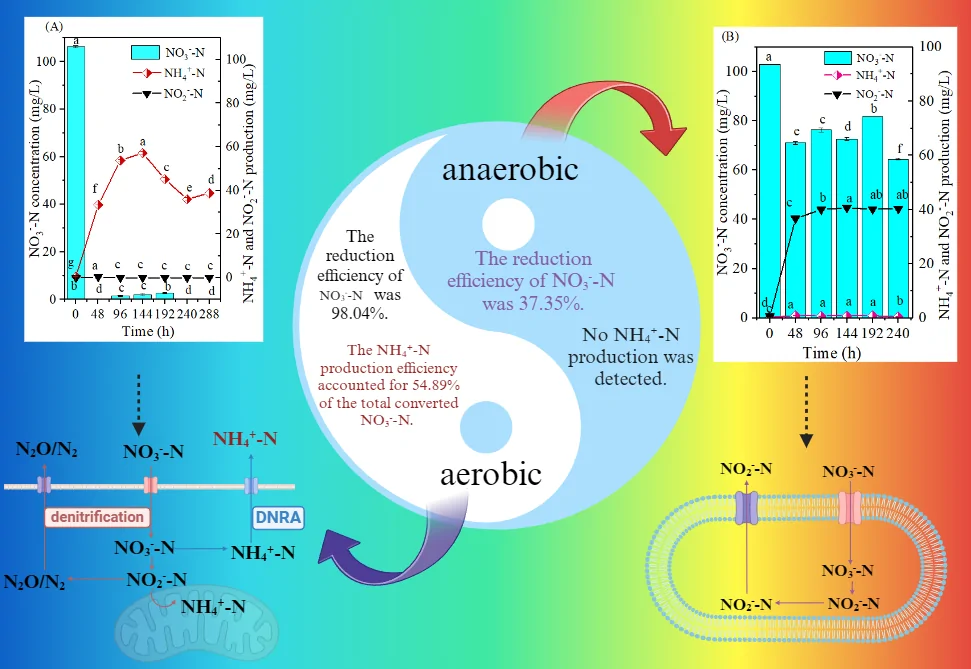
DNRA process of strain EM-F5 under aerobic conditions and nitrate conversion under anaerobic conditions. (**A**) DNRA process of strain EM-F5 under aerobic conditions. (**B**) Nitrate conversion process of strain EM-F5 under anaerobic conditions. Values are expressed as means ± SD for three replicates. Different letters indicate significant differences between treatments at *P* < 0.05.

According to reports, most of the current research on DNRA has been carried out by bacteria under anaerobic conditions. However, DNRA under anaerobic conditions generally has the drawbacks of high cost and strict condition control. Therefore, it is necessary to explore more other metabolic microorganisms that can efficiently perform DNRA under aerobic conditions. To explore the ability and characteristics of actinomyces strain EM-F5 to perform DNRA under aerobic conditions, the content of inorganic nitrogen was detected every 48 h. The results indicated that nitrate was almost completely converted (99.86%) within 48 h (Fig. 2A), indicating that strain EM-F5 could grow and reproduce in the high-nitrate environment, whereas most bacteria currently fail to completely remove nitrate or produce a large number of intermediate products, as is the case for *Pseudomonas mendocina* X49 (23) and *Pseudomonas taiwanensis* EN-F2 (19). Ammonium accumulation increased with the culture duration and peaked at 144 h, accounting for 54.89% of the reduced nitrate. This percentage was much larger than that achieved with DNRA strains of *Pseudomonas putida* Y-9 under aerobic conditions, which produced only 3.42 mg/L and 6.66 mg/L of ammonium, respectively (6. In contrast to the high nitrite yield under anaerobic conditions, no nitrite was detected under aerobic conditions when the strain EM-F5 utilized starch as the carbon source, which is opposite to that observed with *Pseudomonas fluorescens* 2P24 (24). This finding indicated that the rate of nitrite consumption might be greater than or equal to its production rate. The aforementioned results demonstrated that strain EM-F5 was an aerobic DNRA-active strain that offers the benefits of high efficiency, few by-products, low cost, and environmental safety in the DNRA process.

A nitrogen balance analysis of strain EM-F5 in the current system was calculated under aerobic conditions and was presented in Table 2. After 144 h of culture, the strain EM-F5 produced 44.43 ± 1.73 mg/L ammonium, and the remaining nitrate concentration was 5.11 ± 0.21 mg/L. At this time, no nitrite was detected, which further confirmed the previous exploration experiment on the DNRA ability of strain EM-F5 under aerobic conditions. Most of the converted nitrates were transformed to ammonium and intracellular nitrogen (26.52 ± 1.16 mg/L), and the proportion converted to ammonium was larger. Meanwhile, the nitrogen loss was only 25.46%, indicating that strain EM-F5 was mainly involved in the DNRA process under aerobic conditions.

**TABLE 2:** Nitrogen balance of DNRA in strain EM-F5 under aerobic conditions

Initial TN (mg/L)	Final N (mg/L)					Intracellular-N (mg/L)	N loss (%)
	NO_3_^−^-N	NH_2_OH	NO_2_^−^-N	NH_4_^+^-N	Organic-N		
107.48 ± 1.28	5.11 ± 0.21	0	0	44.43 ± 1.73	4.06 ± 0.93	26.52 ± 1.16	25.46%

#### Nitrate reduction under anaerobic conditions

To date, DNRA has been mainly performed by certain facultative and strictly anaerobic bacteria, which are widespread in environments such as wastewater treatment systems, soils, and marine sediments (25). Under anaerobic conditions, DNRA microorganisms reduce nitrate/nitrite by using organic matter or other electron donors (hydrogen, sulfide, or iron). For example, *Bacillus paralicheniformis* LMG6934 produced 0.99, 4.35, and 8.53 mmol/L of ammonium by the reduction of dissimilated nitrite (1.0, 5.0, and 10.0 mmol/L, respectively). The higher the nitrite concentration, the greater the ammonium production (26). To explore whether strain EM-F5 had the ability, using starch as the carbon source, to perform DNRA under anaerobic conditions, the concentrations of nitrate, ammonium, and intermediate metabolites were detected at every 48-h intervals. The nitrate concentration decreased from 102.90 to 64.47 mg/L after culture for 240 h, and the conversion efficiency was 37.35% (Fig. 2B). These values were much lower than those of the anaerobic denitrification strain *Acinetobacter* sp. SZ28, which was isolated from a nutrient-poor reservoir and could reduce nitrate by 76.40% in 8 days (27). Under anaerobic conditions, the ammonium accumulation with strain EM-F5 was only 0.51 mg/L. It was noteworthy that the nitrite concentration increased from 0.62 mg/L at 0 h to 40.32 mg/L at 240 h, indicating that nitrate was mainly converted to nitrite. Therefore, the strain EM-F5 could not perform DNRA under anaerobic conditions but could convert some nitrate.

In order to further verify the nitrogen conversion of strain EM-F5 under anaerobic conditions, the nitrogen balance experiment was continued (Table 3). It could be seen from Table 3 that most of the converted nitrate in the anaerobic conditions was converted to nitrite (22.99 ± 0.17 mg/L) and the accumulation of ammonium was only 2.34 ± 0.13 mg/L, so the nitrogen loss was 0. The enzyme activity of nitrite reductase (NIR) was only 0.004 ± 0.12 U/mg protein. This phenomenon further confirmed that strain EM-F5 could not perform DNRA under anaerobic conditions.

**TABLE 3:** Nitrogen balance of strain EM-F5 under anaerobic conditions

Initial TN (mg/L)	Final N (mg/L)					Intracellular-N (mg/L)	N loss (%)
	NO_3_^−^-N	NH_2_OH	NO_2_^−^-N	NH_4_^+^-N	Organic-N		
110.74 ± 0.58	63.92 ± 1.11	0	22.99 ± 0.17	2.34 ± 0.13	23.08 ± 0.22	1.04 ± 0.05	0

According to the experimental results of anaerobic and aerobic conditions, strain EM-F5 could efficiently complete DNRA under aerobic conditions and realize partial nitrate conversion under anaerobic conditions. This conflicts with the traditional view that most bacteria can only perform DNRA under anaerobic conditions and denitrification under aerobic conditions. Ammonium accumulated to a greater extent in the aerobic environment, which is simpler to manage and operate than anaerobic environments. Most importantly, the aerobic DNRA process may utilize energy from the substrate more effectively, stimulate the growth of microorganisms and facilitate ammonium accumulation, and avoid producing higher-risk intermediate products. Therefore, the high-efficiency ammonium production of strain EM-F5 under aerobic conditions has certain economic value and is worth further study at the molecular level.

### Effects of environmental factors on DNRA capability of strain EM-F5

#### Dissolved oxygen

**Fig 3:**
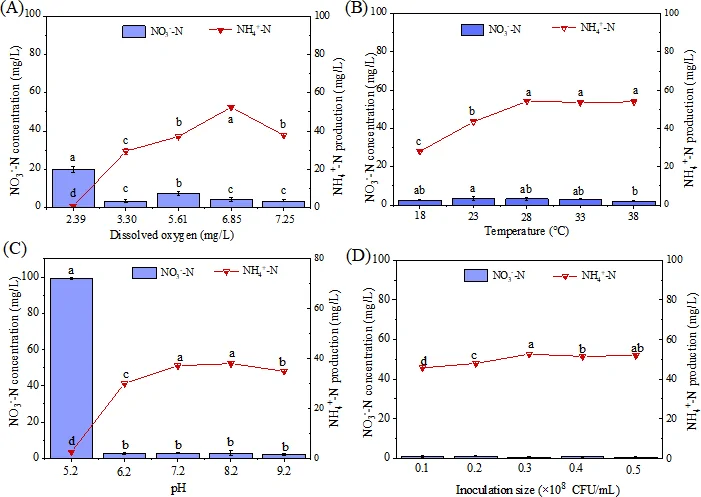
The DNRA abilities and characteristics of strain EM-F5 under different environmental factors. (**A**) Dissolved oxygen; (**B**) temperature; (**C**) pH; (**D**) inoculation size. Values are expressed as means ± SD for three replicates. Different letters indicate significant differences between treatments at *P* < 0.05.

The dissolved oxygen (DO) concentration varied with shaking speed, corresponding to 2.39, 3.30, 5.61, 7.25, and 6.85 mg/L at 0, 50, 100, 150, and 200 rpm, respectively. Facultative aerobic denitrifiers thrive under both aerobic and anaerobic conditions, whereas DNRA microorganisms are typically obligate anaerobes. High DO may, thus, promote the growth of denitrifiers at the expense of DNRA bacteria, resulting in suppressed DNRA activity under aerobic conditions (28). In contrast, strain EM-F5 had a greater capacity for DNRA in the presence of high DO (Fig. 3A). In the range of 2.39–7.25 rpm, the nitrate conversion efficiencies of strain EM-F5 were 81.44%, 96.66%, 92.66%, 96.32%, and 96.57%, respectively, indicating that DO had little effect on nitrate conversion by strain EM-F5. Strain EM-F5 lacked DNRA capacity when DO was 2.39 mg/L. In contrast to anaerobic strain D1, the DNRA pathway of strain EM-F5 was less oxygen-sensitive. In the DO range of 0.60 to 1.0 mg/L, the highest ammonium accumulation was 44.69% (Wang et al., 2024). The DNRA activity of strain EM-F5 under high DO was stronger than that under low DO, similar to that of *Pseudomonas stutzeri* D6 (29). When DO ranged between 3.30 and 7.25 mg/L, the ammonium accumulation of strain EM-F5 was 29.49, 37.22, 52.37, and 37.83 mg/L, respectively, corresponding to percentage ammonium accumulation of 28.86%, 38.55%, 51.80% and 38.74%. The highest ammonium production efficiency was achieved under a DO of 7.25 mg/L. The ammonium production was much higher than that of *Pseudomonas putida* Y-9 (6) and *Streptomyces* sp. XD-11-6-2 (30). Compared with traditional anaerobic DNRA microorganisms, strain EM-F5 may have different aerobic regulatory mechanisms.

#### Temperature

Temperature has a significant effect on DNRA by bacteria. Higher temperatures are generally beneficial to improve microbial activity as well as the abundance and community structure of DNRA microorganisms (31). The DNRA capability of strain EM-F5 at 18, 23, 28, 33, and 38°C was investigated (Fig. 3B). The strain could tolerate high and low temperatures and efficiently perform DNRA in a wide temperature range. After culture of strain EM-F5 for 144 h in simulated wastewater medium, the residual nitrate concentration at each temperature was 2.68, 3.44, 3.22, 3.07, and 2.15 mg/L, respectively, corresponding to conversion efficiencies of 97.30%, 96.77%, 96.75%, 96.96% and 97.73%, respectively. This finding indicated that nitrate conversion was little impacted by temperature in the range tested. Meanwhile, ammonium accumulation increased from 28.05 mg/L at 18℃ to 53.85 mg/L at 38℃, which corresponded to a rise in ammonium yield from 29.48% to 56.70%. The increase in temperature was conducive to the DNRA capacity of strain EM-F5. A possible reason was that the increase in temperature increased the metabolic activity of strain EM-F5 and thus enhanced the DNRA efficiency (32). In this study, at temperatures of 28, 33, and 38℃, the ammonium production efficiency was 55.86%, 56.16%, and 56.70%, respectively. The DNRA efficiency of strain EM-F5 would cease to be promoted with a further increase in temperature, which was inconsistent with the conclusion reported by Lai et al. (33). Their study demonstrated that high temperature would create more reductive conditions for DNRA. Consequently, with the increase in temperature, DNRA was enhanced. Similarly, Wei et al. (13) reported that DNRA occurred between 5℃ and 35℃ and that the DNRA rate increased exponentially with temperature. In conclusion, strain EM-F5 performed DNRA efficiently under a broad range of temperatures.

#### pH

The effect of pH on microorganisms comprises two aspects: on the one hand, the pH can affect the cell surface charge, and, subsequently, the absorption and utilization of nutrients by the microorganisms; on the other hand, the pH can inhibit enzyme activity within microbial cells (34). Different microorganisms differ in adaptability to the environmental pH. Generally, microorganisms can grow and develop within a pH range of 4.0–9.0, but most nitrogen-converting microorganisms show better conversion efficiency under weakly alkaline or alkaline conditions. However, relatively few pertinent studies have examined the effect of pH on DNRA. In this study, the DNRA of strain EM-F5 was assessed at pH values of 5.20, 6.20, 7.20, 8.20, and 9.20. The nitrate conversion efficiency was 3.84%, 97.45%, 97.27%, 97.20%, and 97.73%, and the ammonium accumulation was 2.57, 30.14, 55.13, 48.02, and 44.93 mg/L, respectively, among which the optimal pH was 7.20 (Fig. 3C). Strain EM-F5 exhibited DNRA activity across a broad pH range (6.20–9.20). This broad adaptability might be attributed to the pH-dependent activity profiles of key enzymes: cytochrome c nitrite reductase (NrfA), which was critical for DNRA, showed higher activity at elevated pH, whereas the denitrification enzyme CuNir performed better under acidic conditions. Thus, a higher pH would favor DNRA over denitrification (35). Meanwhile, the ideal reaction rate for non-NrfA type DNRA was at pH 7.50, according to Sorokin et al. (36), indicating that NrfA enzymes may be more active in conditions that are near-neutral or slightly alkaline. Wei et al. (13) observed that DNRA could occur between pH 5.0 and 8.0, but the efficiency was greatest at pH 7.0, which further emphasizes the efficient activity of NrfA enzymes in a neutral environment. Furthermore, no nitrite was detected in the whole optimization pH process, indicating that the rate of nitrite consumption might be greater than or equal to its production rate. The foregoing results showed that strain EM-F5 could perform DNRA across a broad pH range and exhibited high accumulation of ammonium.

#### Inoculum size

Through its effects on microbe concentration, competitive relationship, and nutrient distribution, the inoculum size can indirectly alter the effectiveness and impact of DNRA process. Excessive inoculum results in inadequate nutrition, which will limit the continuous growth of microorganisms and lead to autolysis of the existing microorganisms. However, insufficient inoculum affects the proliferation of microorganisms and reduces the capability for nitrogen conversion (37). In this study, the nitrate conversion efficiency of different inoculum sizes (the initial OD_600_ = 0.10, 0.20, 0.30, 0.40, and 0.50 × 10^8^ CFU/L) was as high as 99.08%, 98.87%, 99.50%, 99.62%, and 99.38%, respectively, after culture for 144 h (Fig. 3D). The strain EM-F5 could convert a high concentration of nitrate (100 mg/L) in the inoculum range of 0.10–0.5 × 10^8^ CFU/L. The ammonium accumulation was 45.70, 53.58, 58.96, 51.78, and 52.28 mg/L, respectively. The optimal inoculum concentration was 0.30 × 10^8^ CFU/L, but no nitrite was detected throughout the culture period. In conclusion, strain EM-F5 could effectively perform DNRA in the inoculum range of 0.10–0.5 × 10^8^ CFU/L.

#### S^2−^/NO_3_^−^

**Fig 4:**
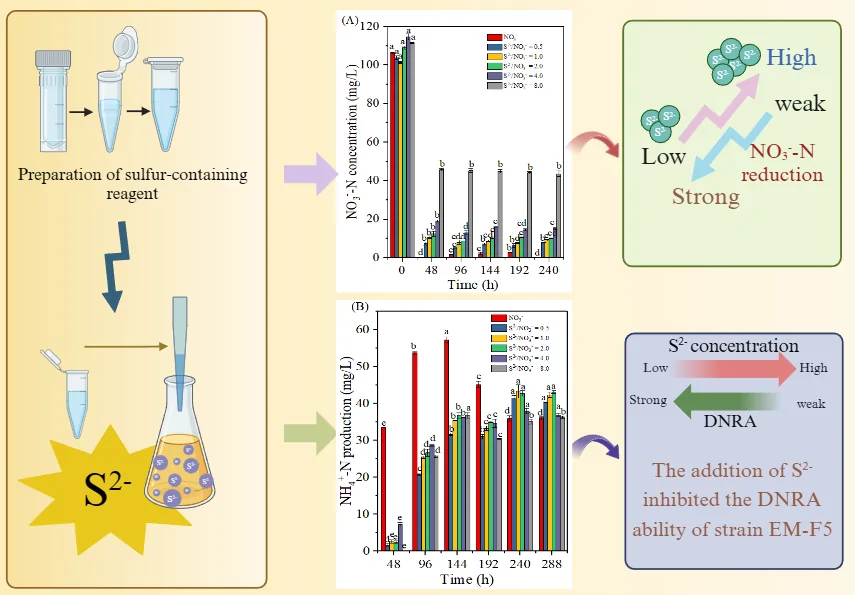
Effects of different S^2−^/NO_3_^−^ on aerobic DNRA of strain EM-F5. (**A**) NO_3_^−^-N concentration; (**B**) NH_4_^+^-N production. Values are expressed as means ± SD for three replicates. Different letters indicate significant differences between treatments at *P* < 0.05.

Sulfides can affect intracellular electron transfer by interacting with microbial cytochromes and ferredoxins, thereby affecting DNRA. To explore the effects of different sulfides on DNRA, the S^2**−**^/NO_3_**^−^** ratio was set to 0.50, 1.0, 2.0, 4.0, or 8.0, respectively. After culture for 48 h, nitrate had been completely transformed in the medium without the addition of S^2**−**^ (Fig. 4), whereas residual nitrate concentrations of 7.29, 10.49, 12.36, 18.98, and 45.69 mg/L, respectively, remained after addition of different concentrations of S^2−^. The results indicated that the addition of S^2−^ inhibited the conversion of nitrate by strain EM-F5, and the degree of inhibition increased with the increase in sulfur concentration. This phenomenon differed from the study of Zheng et al. (38), in which S^2−^ as an electron donor promoted the reduction of nitrate to ammonium by *Rhodobacteraceae*, *Arcobacter*, *Sulfurospirillum,* and *Denitrovibrio* microorganisms. A possible reason was that the presence of sulfide could activate sulfur-metabolizing bacteria, thus promoting their utilization of nitrogen sources and ultimately increasing ammonia production. With extension of the culture period to 144 h, ammonium production without addition of S^2−^ peaked (at 57.19 mg/L) at this time point, and the ammonium production efficiency was 58.85%, which was higher than that of chemical autotrophic *Thioploca* bacteria (48%) (39). After the addition of different concentrations of S^2−^, ammonium accumulation was relatively constant at approximately 35.0 mg/L, which still represented an inhibitory effect. At this time, the residual nitrate concentration was 2.08, 6.96, 8.70, 10.23, 15.93, and 44.80 mg/L, which may be associated with SO_4_^−^ generated by the transformation of S^2−^. The SO_4_^−^ would compete for electrons needed for NO_3_^−^ conversion (40), resulting in a decrease in DNRA efficiency. At the end of the culture period (240 h), ammonium accumulation in the medium lacking S^2−^ decreased to 35.99 mg/L, whereas with an increase in the S^2−^/NO_3_^−^ ratio from 0 to 0.50 and 1.0, ammonium accumulation increased to 41.52 and 43.30 mg/L, respectively, indicating the transient inhibitory effect of a low S^2−^/NO_3_^−^ ratio. However, after 288 h of culture, the DNRA ability of the strain was promoted. With an increase in the S^2−^/NO_3_^−^ ratio from 1.0 to 8.0, although the reaction time was prolonged, an inhibitory effect was still observed. A possible reason was that a high sulfide concentration had a toxic effect on the microorganism and inhibited microbial activity. This inhibition was similar to that reported for *Pseudomonas*. LZ-1 (41).

#### Fe^2+^/NO_3_^**−**^

**Fig 5:**
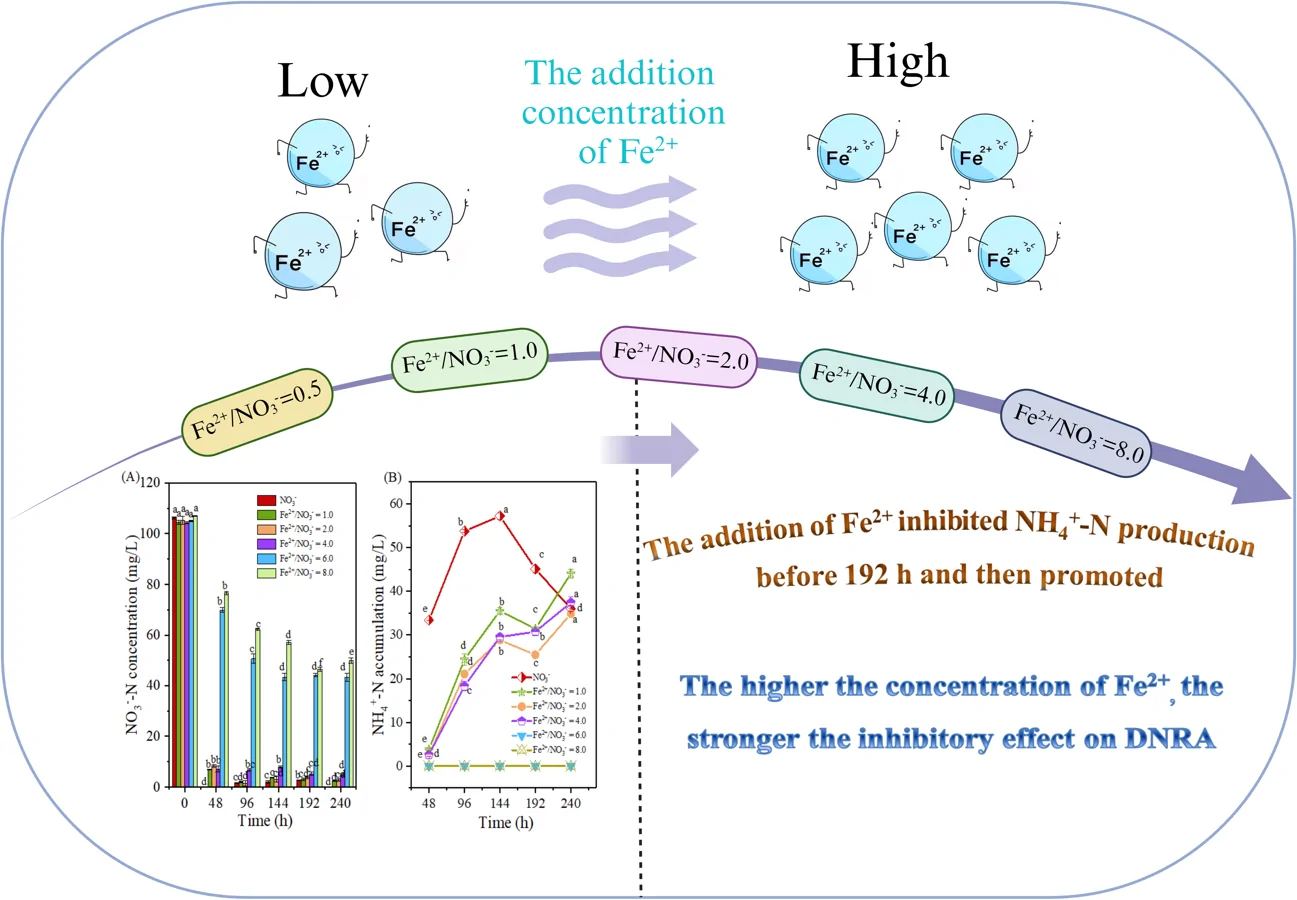
Effects of different Fe^2+^/NO_3_^−^ on aerobic DNRA of strain EM-F5. (**A**) NO_3_^−^-N concentration; (**B**) NH_4_^+^-N production. Values are expressed as means ± SD for three replicates. Different letters indicate significant differences between treatments at *P* < 0.05.

Iron has been implicated in critical mechanisms, such as cell respiration, tricarboxylic acid cycling, ribosome assembly and recycling, oxidative stress tolerance, and DNA synthesis and damage repair (42). Iron can directly affect the growth and metabolism of DNRA microorganisms and participate in electron transport and energy generation. To study the effect of iron on the newly isolated actinomycete strain EM-F5, nitrate was used as the nitrogen source and the Fe^2+^/NO_3_^−^ ratio was set to 1.0, 2.0, 4.0, 6.0, or 8.0. Ammonium accumulation and nitrate conversion efficiency both declined with the addition of varying Fe^2+^ concentrations to the medium (Fig. 5). At 48 h, the nitrate in the medium lacking Fe^2+^ was completely transformed, whereas residual nitrate of 6.77, 8.34, 7.08, 69.70, and 76.57 mg/L, respectively, remained after the addition of different concentrations of Fe^2+^. At this time point, the addition of Fe^2+^ inhibited nitrate transformation, and the degree of inhibition increased with elevation in the Fe^2+^ concentration. Most previous studies have shown that the presence of Fe^2+^ could promote DNRA. For example, assessment of DNRA with Fe^2+^ as the electron donor in river sediments indicated that when the Fe^2+^ concentration in the sediment reaches its peak (>400 µmol/L), the sediment changed from denitrification to DNRA under oxidation conditions (32). In paddy soil, the increase in Fe^2+^ concentration significantly enhanced the efficiency of DNRA (43). Most studies suggested that DNRA with Fe^2+^ as the electron donor was performed by chemoautotrophic or heterotrophic or mixotrophic microorganisms. Molecular studies had identified a variety of microbial strains that mediated oxidative coupling of NO_3_^−^ and Fe^2+^ (44). However, these studies provided no evidence of denitrification coupling with Fe^2+^. In this study, the addition of Fe^2+^ could inhibit DNRA by strain EM-F5, possibly because the regulatory genes differ from those previously reported. With extension of the culture period to 144 h, the ammonium accumulation in the medium lacking Fe^2+^ peaked (at 57.19 mg/L), and the ammonium production efficiency was 54.89%. The ammonium concentration in the medium supplemented with different concentrations of Fe^2+^ was 35.51, 28.85, 29.55, 0, and 0 mg/L. Thus, although the reaction time was prolonged, an inhibitory effect was still observed, and the nitrate concentration was 2.08, 3.65, 3.03, 7.92, 43.41, and 57.04 mg/L. Nevertheless, strain EM-F5 possessed DNRA competence when Fe^2+^/NO_3_^−^< 4 and the ammonium output was maintained at approximately 35 mg/L. Under Fe^2+^/NO_3_^−^ ratios of 6 and 8, the nitrate conversion efficiency was 58.66% and 53.39%, respectively, although no ammonium was produced. The foregoing results suggested that a low Fe^2+^/NO_3_^−^ ratio was beneficial for DNRA and high Fe^2+^/NO_3_^−^ was beneficial to denitrification. This phenomenon differed from the conclusion that a high concentration of Fe^2+^ can disrupt intracellular electron transport and inhibit denitrification, thus promoting the DNRA process (45). Further exploration at the molecular level is needed to understand the inhibitory mechanism of Fe^2+^ on the aerobic DNRA process of strain EM-F5.

#### NO_2_^−^/NO_3_^−^

**Fig 6:**
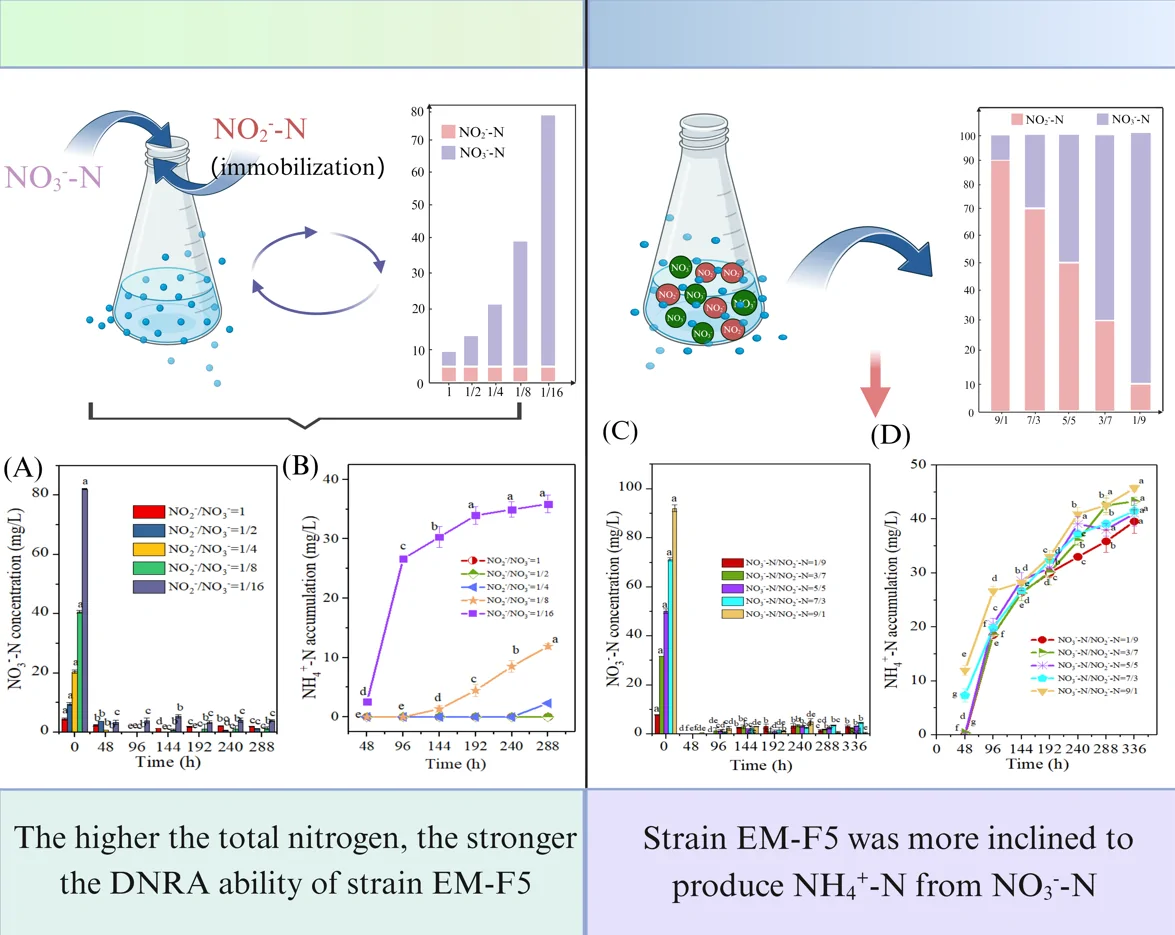
Effects of different NO_2_^−^/NO_3_^−^ on aerobic DNRA of strain EM-F5. (**A**) NO_3_^−^-N concentration with total nitrogen change; (**B**) NH_4_^+^-N production with total nitrogen change; (**C**) NO_3_^−^-N concentration without total nitrogen change; (**D**) NH_4_^+^-N production without total nitrogen change. Values are expressed as means ± SD for three replicates. Different letters indicate significant differences between treatments at *P* < 0.05.

Both NO_2_**^−^** and NO_3_**^−^** are usually present in the soil and water, and the NO_2_**^−^**/NO_3_**^−^** ratio is a critical factor that determines the choice of the pathway (denitrification and DNRA) and affects the distribution of reduction products. Previous studies have shown that the crucial factor affecting the competition between DNRA and denitrifying bacterial communities is not the relative supply of NO_2_**^−^** and NO_3_**^−^** but the ratio between NO_2_**^−^** and NO_3_**^−^** (46). To explore the influence of the NO_2_**^−^**/NO_3_**^−^** ratio on DNRA, the initial nitrite concentration was fixed at 5.0 mg/L, and the nitrate concentration was set to 5.0, 10.0, 20.0, 40.0, and 80.0 mg/L, resulting in NO_2_**^−^**/NO_3_**^−^** ratios of 1, 1/2, 1/4, 1/8, and 1/16, respectively (Fig. 6). With increasing nitrate addition from 5.0 to 80.0 mg/L, the nitrate conversion efficiency after 48 h increased progressively, reaching 46.55%, 60.59%, 95.94%, 100%, and 95.90%, respectively. At this time point, no ammonium was detected. The denitrification process may be the primary cause of the lack of DNRA capacity under a high NO_2_^−^/NO_3_^−^ ratio, as evidenced by the absence of ammonium under an NO_2_^−^/NO_3_^−^ ratio of 1 and 1/2 throughout the culture period of 288 h. Only 2.30 mg/L of ammonium was generated under an NO_2_^−^/NO_3_^−^ ratio of 1/4. With an increase in total nitrogen, i.e., under NO_2_^−^/NO_3_^−^ ratios of 1/8 and 1/16, the ammonium accumulation was 11.89 and 35.88 mg/L, and the ammonium production efficiencies were 35.72% and 47.16%, respectively. This phenomenon was contrary to the conclusion that respiratory ammoniation plays a leading role in *Shewanella loihica* PV-4 at a high NO_2_^−^/NO_3_^−^ ratio, whereas a low NO_2_^−^/NO_3_^−^ ratio was conducive to denitrification (46). We speculated that the DNRA regulatory gene of strain EM-F5 may differ from that of bacteria previously reported. Thus, strain EM-F5 did not possess DNRA ability under NO_2_^−^/NO_3_^−^ > 1/4, i.e., when NO_2_^−^ was 5.0 mg/L and NO_3_^−^ < 40.0 mg/L. The DNRA ability of strain EM-F5 was improved under a low NO_2_^−^/NO_3_^−^ ratio, meaning that the amount of nitrogen in the system increased.

To further explore the differences in nitrogen utilization of strain EM-F5 under mixed NO_3_^−^ and NO_2_^−^ conditions, the effects of different NO_2_^−^/NO_3_^−^ ratios on DNRA while keeping total nitrogen unchanged were investigated. The NO_3_^−^/NO_2_^−^ ratio was set to 1/9, 3/7, 5/5, 7/3, and 9/1 to determine the DNRA ability of strain EM-F5. Regardless of the NO_2_^−^/NO_3_^−^ ratio, the ammonium accumulation displayed an increasing trend (Fig. 6C and D). The maximum ammonium accumulated for each ratio was 39.55, 43.28, 41.02, 41.52, and 45.73 mg/L after culture for 336 h, indicating that strain EM-F5 could produce ammonium efficiently under the conditions of mixed nitrogen sources in different proportions. Compared with *Pseudomonas* sp. XS-18, which did not produce ammonium in mixed NO_3_^−^ and NO_2_^−^ environments (47), strain EM-F5 had greater advantages and was more conducive to DNRA in a broad range of mixed-nitrogen source environments. In addition, in the mixed system, the ammonium production of strain EM-F5 increased with an elevation in NO_3_^−^ concentration (i.e., NO_3_^−^/NO_2_^−^ ratio), indicating that the strain EM-F5 was more inclined to take advantage of NO_3_^−^ for DNRA. This situation was similar to that in which the DNRA capacity of *Shewanella loihica* PV-4 decreased as the NO_2_^−^ concentration increased (46). However, in contrast to the findings reported by Wang and Gunsalus (48) at a high NO_2_^−^/NO_3_^−^ ratio, the NO_3_^−^ concentration could induce and regulate the expression of the NO_2_^−^ reductase-related *nrfA* gene in this pathway, which was conducive to the conclusion of the DNRA pathway. One possible reason was that strain EM-F5 lacked the *nrfA* gene or the genes regulating DNRA differ. In conclusion, strain EM-F5 preferentially used nitrate for DNRA when NO_3_^−^ and NO_2_^−^ were present simultaneously, and nitrate was the primary source of the ammonium generated.

### Physiological activity analysis of strain EM-F5 during aerobic DNRA

Research indicated that the ETSA could reflect the activity of respiratory enzymes and the status of electron transfer in microorganisms (49). ATP served as the primary energy carrier in cells and directly influenced microbial physiological activity and metabolic capacity. Meanwhile, the NADH/NAD^+^ ratio modulated the intracellular metabolic state, redox balance, and energy metabolism activity (50). Investigating the DNRA in *Streptomyces* from a physiological activity perspective could further demonstrate its high efficiency.

**Fig 7:**
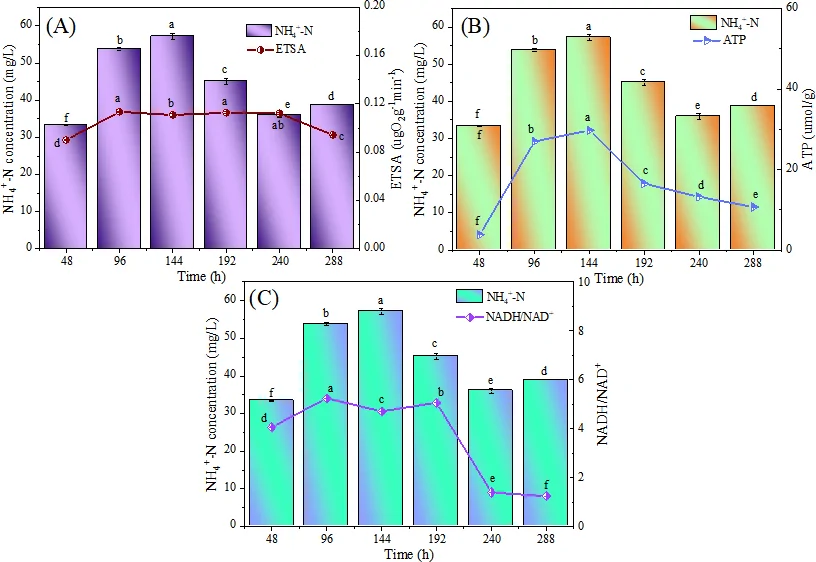
Physiological activity indicators of the strain EM-F5 for aerobic DNRA. (**A**) Production performances of ETSA and NH_4_^+^-N. (**B**) Production performances of ATP and NH_4_^+^-N. (**C**) Production performances of NADH/NAD^+^ and NH_4_^+^-N. Values are expressed as means ± SD for three replicates. Different letters indicate significant differences between treatments at *P* < 0.05.

Strain EM-F5 was cultured in aerobic DNRA medium, and ETSA, ATP, and NADH/NAD^+^ were detected at intervals of 48 h. The result was shown in Fig. 7. The ETSA reached its peak (0.1134 ugO_2_g^−1^min^−1^) at 96 h. The consistently high ETSA values (mostly > 0.110 ugO_2_g^−1^min^−1^ from 96 to 240 h) ensured rapid and efficient electron transfer from initial donors to nitrate reductase, thereby effectively driving the reductive reaction. As the direct energy currency, ATP levels showed a significant positive correlation with ammonium production. From the early to peak phase (48–144 h), the ATP concentration increased sharply from a very low level of 3.84 to a peak of 29.73 umol/g, representing a more than sevenfold increase. Concurrently, ammonium production increased from 33.37 mg/L to its maximum of 57.19 mg/L. This clearly indicated that higher intracellular ATP content reflected greater cellular metabolic activity (51), and the acceleration of the DNRA process strongly depended on elevated cellular energy status. The high ATP levels (maintained between 27.02 and 29.73 umol/g from 96 to 144 h) provided sufficient energy for processes such as nitrate transmembrane transport and reductase synthesis (52). Furthermore, the NADH/NAD^+^ ratio reached its highest value of the entire cycle (5.24) when DNRA efficiency increased rapidly at 96 h, indicating substantial accumulation of cellular reducing power. DNRA was a multistep reductive process that required several electrons (typically supplied by NADH) to reduce one nitrate molecule to ammonium. Therefore, the high NADH/NAD^+^ ratios during the peak phase (maintained between 4.71 and 5.24 from 96 h to 192 h) directly ensured the material basis for high ammonium yield. Together, these results demonstrated that strain EM-F5 regulated aerobic DNRA by coordinating its electron transport system, energy metabolism, and redox balance.

### Analysis of enzyme activity related to aerobic DNRA

The aerobic DNRA carried out by microorganisms depends on the action of related enzymes. NR and NIR catalyze the reduction of nitrate and nitrite. To investigate the DNRA nitrogen metabolism characteristics of strain EM-F5 more comprehensively, the specific activities of NR and NIR of strain EM-F5 were determined. The results were shown in Table 4. The specific activities of NR and NIR in the aerobic DNRA performed by strain EM-F5 were 0.323 and 0.018 U/mg protein, respectively. The specific activity of NR was significantly higher than that of 0.02 U/mg protein in strains *P. putida* NP5 and *Paracoccus denitrificans* ISTOD1 (53, 54). In addition, the NIR specific activity of strain EM-F5 was similar to that of strains *Klebsiella pneumoniae* CF-S9 (0.02 U/mg protein) and *Diaphorobacter* sp. PD-7 (0.019 U/mg protein) (55, 56) but lower than that of *Streptomyces mediolani* EM-B2 (0.024 U/mg protein)(57). This might be the fundamental reason why the ammonium production of strain EM-F5 was lower than that of strain EM-B2. The detection of the specific activities of NR and NIR enzymes indicated that this enzyme might exist in the strain EM-F5 and catalyze the metabolic process of DNRA in this bacterium.

**TABLE 4:** Specific activity of related enzymes in aerobic DNRA of strain EM-F5

Enzyme	Specific activity (U/mg protein)
NR	0.323 ± 0.05
NIR	0.018 ± 0.12

### Conclusions

The novel actinomycete *Streptomyces hydrogenans* EM-F5 was isolated from the Caohai Wetland, which expanded the known repertoire of aerobic DNRA-capable strains. The reduction of nitrate was basically completed by strain EM-F5 under aerobic conditions, and the production efficiency of ammonium accounted for 54.89% of the converted nitrate. Anaerobically, strain EM-F5 converted 37.35% of nitrate, partly to nitrite rather than via complete DNRA. In addition, strain EM-F5 could perform DNRA efficiently under a broad range of environmental conditions. Compared with nitrite, strain EM-F5 was more inclined to utilize nitrate for DNRA. The detection of enzyme specific activity, nitrogen balance, and physiological activity indicators further demonstrated the high efficiency of strain EM-F5 in performing aerobic DNRA. This study provides basic data for the improvement of ammonium production through DNRA and offers a promising candidate for nitrogen retention and recycling in agricultural soils.

## Data Availability

The full-length 16S rRNA gene sequence of *Streptomyces hydrogenans* strain EM-F5 was submitted to GenBank under accession number PQ386394. The original contributions presented in the study are included in the article; further inquiries can be directed to the corresponding authors.
